# Measuring Basic Reproduction Number to Assess Effects of Nonpharmaceutical Interventions on Nosocomial SARS-CoV-2 Transmission

**DOI:** 10.3201/eid2807.212339

**Published:** 2022-07

**Authors:** George Shirreff, Jean-Ralph Zahar, Simon Cauchemez, Laura Temime, Lulla Opatowski, EMEA-MESuRS Working Group on the Nosocomial Modelling of SARS-CoV-2

**Affiliations:** Institut Pasteur, Université Paris Cité, Paris, France (G. Shirreff, S. Cauchemez, L. Opatowski);; Conservatoire National des Arts et Métiers, Paris (G. Shirreff, L. Temime);; University of Versailles Saint-Quentin-en-Yvelines, Montigny-le-Bretonneux, France (G. Shirreff, L. Opatowski);; Assistance Publique–Hôpitaux de Paris, Bobigny, France (J.-R. Zahar);; PACRI Unit, Paris (L. Temime)

**Keywords:** COVID-19, respiratory infections, severe acute respiratory syndrome coronavirus 2, SARS-CoV-2, SARS, coronavirus disease, zoonoses, viruses, coronavirus, nosocomial infection, patient-to-patient transmission, long-term care facility, transmission rate, basic reproduction number, R_0_, contact precautions, stochastic modelling, statistical inference, iterative filtering

## Abstract

Outbreaks of SARS-CoV-2 infection frequently occur in hospitals. Preventing nosocomial infection requires insight into hospital transmission. However, estimates of the basic reproduction number (R_0_) in care facilities are lacking. Analyzing a closely monitored SARS-CoV-2 outbreak in a hospital in early 2020, we estimated the patient-to-patient transmission rate and R_0_. We developed a model for SARS-CoV-2 nosocomial transmission that accounts for stochastic effects and undetected infections and fit it to patient test results. The model formalizes changes in testing capacity over time, and accounts for evolving PCR sensitivity at different stages of infection. R_0_ estimates varied considerably across wards, ranging from 3 to 15 in different wards. During the outbreak, the hospital introduced a contact precautions policy. Our results strongly support a reduction in the hospital-level R_0_ after this policy was implemented, from 8.7 to 1.3, corresponding to a policy efficacy of 85% and demonstrating the effectiveness of nonpharmaceutical interventions.

Despite sweeping control measures, SARS-CoV-2 continues to pose a major threat to older persons and persons with comorbidities, both of whom can have poorer clinical outcomes (*1*,*2*). Thus, hospitals and long-term care facilities (LTCFs) must be particularly vigilant to prevent the spread of SARS-CoV-2 infection among their patients. Nosocomial spread has been an issue since the pandemic began in 2020, and many outbreaks have occurred in hospitals and healthcare facilities, often with high attack and mortality rates (*3*).

To control nosocomial spread, healthcare facilities have progressively implemented preventive measures, such as generalized masking, testing campaigns among patients and staff, isolation, visitor restrictions (*3*), and more recently vaccination (*4*). However, the risk for viral transmission among hospital patients and staff and the effectiveness of control measures remain unclear, and outbreaks still occur (*3*,*5*,*6*).

The basic reproduction number (R_0_) refers to the number of secondary infections caused by a single index infection in an otherwise susceptible population. R_0_ has been widely used as an indicator of SARS-CoV-2 epidemic risk and has also proved valuable for evaluating testing strategies and other preventive measures within healthcare settings (*7*,*8*). R_0_ likely varies between types of healthcare facilities and differs considerably from estimates in the general community (*9*). However, estimating R_0_ in healthcare settings is more challenging than estimating R_0_ in the community. The populations in institutions are small and epidemics are highly stochastic. More data usually are available from hospitals or wards that have more cases. Healthcare facilities rarely test patients randomly or at multiple times during their hospitalizations. Most available data from hospital outbreaks consist of distributions of positive tests over time in a context of evolving testing policy and capacity. 

At the beginning of the pandemic, most countries had no standard strategy or recommendation on how surveillance should be carried out and tests distributed. Testing was mostly conducted on symptomatic patients, and surveillance consisted of possible contact tracing around detected cases. However, unreported asymptomatic cases could represent a substantial fraction of transmissions, and little data on the testing policy are available to estimate how many cases fell through the gaps.

Here, we propose a new framework to analyze detailed hospital test data by using a stochastic transmission model explicitly accounting for testing policy. We estimated R_0_ in the context of a large SARS-CoV-2 outbreak in a LTCF. The outbreak had a high initial R_0_, and we reconstructed the unobserved epidemic to assess effectiveness of nonpharmaceutical interventions.

## Methods

### Hospital and Patient Information

Available data came from a LTCF in Paris, France. The hospital has 3 buildings (A, B, and C), each of which has 4 floors (0–3) that we considered as separate wards. The results of all valid PCR tests were available for each patient identification number during March 1–April 30, 2020 (61 days). Patient information also included the ward to which they were admitted or transferred, admission and discharge dates, and any symptoms they had at first positive test. All dates we provide are relative to the date of the first positive sample in the facility. We censored the data from day 51 onward because the hospital began to change the containment policy after that point. We excluded 23 patients from any ward-level analysis because the ward in which they were tested was unknown (Appendix). We only used anonymized, aggregated patient data and did not collect additional patient data beyond those for clinical use. The Comité Local d’Ethique pour la Recherche Clinique des HUPSSD Avicenne-Jean Verdier-René Muret approved the study as protocol no. CLEA-2021-190. 

### Laboratory Testing

The LTCF collected all nasopharyngeal swab samples from patients. Reasons for testing included having symptoms characteristic of SARS-CoV-2, having had contact with a positive case, or patient transfer between wards or into or out of the hospital (Appendix).

### Model Description

**Figure 1:**
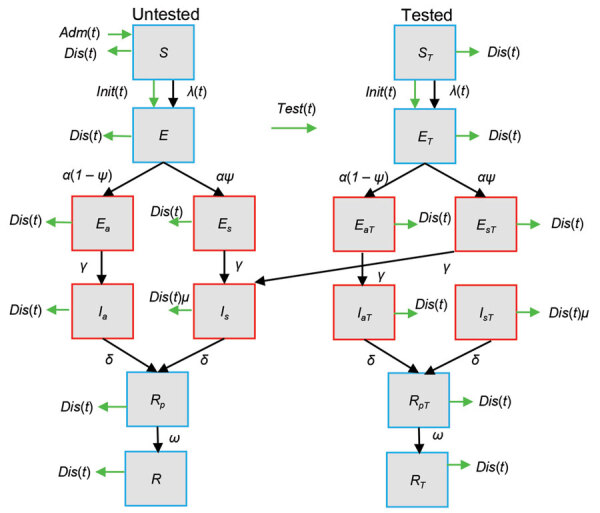
Compartmental susceptible-exposed-infectious-recovered model used to estimate nosocomial SARS-CoV-2 transmission rates on the basis of data for a long-term care facility in France. Red boxes indicate SARS-CoV-2 infectious compartments and blue boxes indicate noninfectious compartments. The left side shows the trajectory of untested persons, the right side shows tested persons. If untested persons are tested at any point in state *X*, they will enter the equivalent tested compartment (*X_T_*, right panel), which is epidemiologically identical except for the testing rate. Patients in the susceptible state (*S*) can become infected by contact with infectious patients. When infected, patients move to the noninfectious incubation (*E*) compartment, after which they can either enter an asymptomatic or a symptomatic pathway of infectiousness. Each pathway has an infectious incubation period (*E_a_*, *E_s_*) before asymptomatic (*I_a_*) or symptomatic (*I_s_*) infection begins. After full infection, patients recover into a noninfectious state (*R_p_*) where they are still likely to test positive before full recovery (*R*) when the probability of testing positive diminishes to (1 – test specificity). Green arrows refer to processes, initiation (*Init*), admission (*Adm*), discharge (*Dis*), and testing (*Test*), that occur a specified number of times on a given day according to model inputs. Black arrows indicate processes that are natural for infection and are entirely stochastic (Appendix Methods, Figure 1). *E*, exposed; *E_a_*, asymptomatic exposed; *E_aT_*, asymptomatic exposed and tested; *E_s_*, symptomatic exposed; *E_sT_*, symptomatic exposed and tested; *E_T_*, exposed and tested; *I*, infectious; *I_a_*, asymptomatic infectious; *I_aT_*, asymptomatic infectious and tested; *I_s_*, symptomatic infectious; *I_sT_*, symptomatic infectious and tested; *I_T_*, infectious and tested; *R*, recovered; *R_p_*, recovered to noninfectious state; *R_pT_*, recovered to noninfectious state and tested; *R_T_*, recovered and tested; *S*, susceptible; *t*, time; α, rate of progression from noninfectious incubation; *ψ*, proportion of patients entering symptomatic pathway; *λ*(*t*), force of infection at time *t*; γ, rate of progression from infectious incubation; *δ*, rate of progression from symptomatic infection; *μ*, relative rate of discharge for symptomatic patients relative to any nonsymptomatic patient; *ω*, rate at which viral shedding ceases during recovery.

We modeled the spread of infection within the LTCF population by using a modified stochastic susceptible-exposed-infected-recovered model (Figure 1; Appendix, Table 1). We defined the force of infection at a given time, *λ*(*t*), as the per-capita rate at which susceptible persons become infected, which we determined by the transmission rate, *β*, and the proportion of infectious patients at that time (Appendix). On the date the epidemic began (*t_init_*), we considered a specific number (*E_init_*) of persons infected. We assumed persons in infectious incubation had reduced infectiousness by a factor of *ε*, compared with symptomatic infected persons. Similarly, we assumed asymptomatic infectious persons had lower infectiousness by a factor of *κ_1_*.

To fully determine transmission over the outbreak period, we compared 2 distinct models. In the primary model, we assumed a single transmission rate, *β*, throughout the study period. However, based on knowledge of changing practices within the hospital, we defined a more complex, 2-phase model in which each phase had its own transmission rate, *β_1_* and *β_2_*, and was delimited by an inflection date, *t_inflect_*. Potential values for *t_inflect_* ranged from day 1, which was the date of the first positive sample, through day 16, which was >1 week after the facility introduced contact precautions and France implemented a generalized lockdown.

We directly computed R_0_ for each stage of infection from the transmission rate, duration of each infectious stage, and the probability infected persons would become symptomatic (Appendix). For the 2-phase model, we computed the average R_0_ by weighting each phase by its duration (Appendix).

### Observation Model

Because of asymptomatic infections, imperfect test sensitivity, and irregular availability of tests, the facility could not identify all infected patients. To account for the imperfect reporting, we added an observation model to the transmission model (Appendix, Figure 1). The observation model assumes all persons are initially untested, but upon testing, the model moves them to an equivalent tested state. Any patient can be retested in the model, but retesting occurs at a reduced relative rate, *ϕ*, estimated directly from the number of tests and retests in the available data (Appendix). When a person in the model develops symptoms, they lose their tested status and rejoin the untested compartment, *I_s_* (Figure 1), enabling the model to account for increased testing when symptoms appear in a patient. However, testing does not change the rates of infectiousness or disease progression.

**Figure 2:**
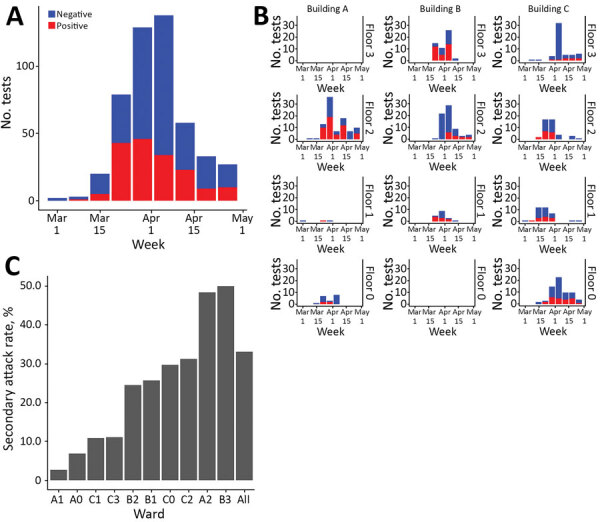
Hospital data from a long-term care facility in France used to estimate nosocomial SARS-CoV-2 transmission rates. A) Number of SARS-CoV-2 PCR tests performed each week in the whole hospital. B) Number of SARS-CoV-2 PCR tests performed in each ward each week. C) Secondary attack rates in the whole hospital. Rates were calculated as the ratio of the number of patients with positive results to the total number of patients in the hospital at any time during the study period.

We used hospital data on the number of admissions, discharges, and tests per day as inputs (Appendix, Figure 2). The model considers admitted patients are in a susceptible untested state and are discharged at random from any state with a relative rate, *μ*, for symptomatic patients. For any day that tests are performed, the model prioritizes patients who have not been tested since becoming symptomatic and conducts any remaining tests at random on the rest of the population (Appendix, Figure 1). We used the sensitivity and specificity of the PCR test at the stage of infection to determine whether patients test positive or negative for SARS-CoV-2.

### Statistical Inference

We calculated the likelihood by comparing the observed numbers of positive and negative cases on each day with the expected numbers generated by the internal model state via the observation process, assuming a binomial distribution (Appendix). We used iterative filtering in the pomp package (*10*) in R (R Foundation for Statistical Computing, https://www.r-project.org) to estimate parameters. In addition to estimating transmission rates, *β*, or *β_1_* and *β_2_*, we also estimated the virus introduction time, *t_init_*, and fixed the initial number of infections, *E_init_*, to 1. For each analysis comprising the same model, dataset, and fixed parameter values, we used profile likelihood to calculate 95% CI for the estimated parameters (Appendix). We compared models by calculating the Akaike information criterion (AIC).

### Model Inference Validation

As a preliminary step, we tested the model and inference methodology on synthetic data. We used this test to ensure that known simulated transmission rates (*β,* or *β_1_* and *β_2_*) and *t_init_* could be recovered by statistical inference (Appendix).

### Hospital- and Ward-Level Analyses 

We first analyzed data at the hospital level, assuming homogeneous mixing across all buildings and wards. We then analyzed the data and estimated parameters for each ward separately. After parameter estimation, we conducted simulations of the visible and undetected parts of the epidemic at both the hospital and ward levels (Appendix).

### Sensitivity Analysis and Time-Varying Reproduction Number

We conducted a sensitivity analysis to identify parameters with variations that most affected our estimated parameters. We perturbed the input parameters, using the lower and upper bound of the CI reported in the literature, and replicated the analysis. For comparison, we used incident cases to calculate the time-varying reproduction number (R_t_) across the entire hospital by using the EpiEstim package (https://CRAN.R-project.org/package=EpiEstim) (Appendix).

## Results

A total of 459 patients were in the hospital during the study period. PCR testing began on day −6; we consider day 1 as the first positive sample was collected. By the end of day 50, 152/312 patients sampled tested positive (Figure 2, panels A, B). The secondary attack rate differed substantially between wards (Figure 2, panel C), ranging from 3% to 50%, and the overall secondary attack rate was 33%.

### Model Inference Validation Results

The results of the validation of parameter inference on synthetic data suggest that sufficient power was available at the hospital level to recover parameters with relatively good accuracy (Appendix, Figures 4, 5). However, power was not always sufficient at the ward level, and we restricted our subsequent analysis of wards to only those where the recovered estimates did not deviate excessively in the estimates of *β* (Appendix Figures 6,7).

### Whole-Hospital Analysis

**Figure 3:**
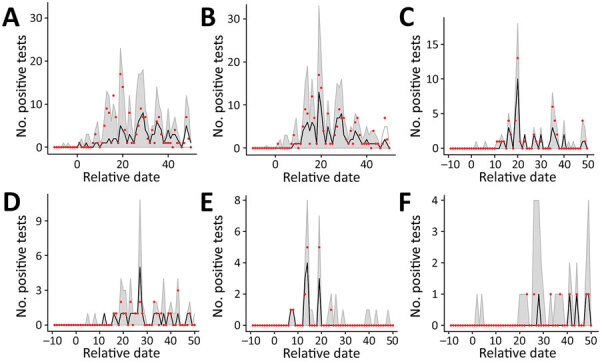
Results of simulated epidemics in a model of nosocomial SARS-CoV-2 transmission using estimated parameters determined on the basis of data from a long-term care facility in France. A) 1-phase model for the whole hospital. B) 2-phase model for the whole hospital. C–F) 1-phase model for individual wards: A2 (C), C0 (D), C2 (E), and C3 (F). Red dots show the observed number of positive tests in the data, black dashed lines indicate the median across that date for all simulations, and gray shading indicates the 95% CI range of the simulated values. Input parameter sets were included if their likelihood fell within the 95% CI relative to the maximum likelihood for 1- and 2-phase models for the whole hospital and individual wards. Estimated parameters are from Tables 1, 2. Extinct epidemics (i.e., those having <3 cumulative cases) were excluded from the distribution.

We calculated estimations of transmission rates at the whole hospital level (Table 1; Appendix). In the 2-phase model, using day 12 as *t_inflect_* gave the best model fit (Appendix Table 4), which is 6 days after the facility officially introduced an obligatory mask-wearing policy and cancellation of all group activities between patients. This model proved a better fit to the data than the 1-phase model, as measured by the AIC (Table 1). Simulated curves from the observed epidemic produced by the models show that the 2-phase model captured the early peak in cases better than the 1-phase model (Figure 3, panels A, B).

**Table 1:** Best estimates and ranges for parameters from 2 models applied to hospital data from a long-term care facility in France to estimate nosocomial transmission rates of SARS-CoV-2*

Parameter	Model	
	1-phase	2-phase
*β*	0.38 (0.30–0.60)	NA
* β_1_*	NA	1.28 (0.76–2.40)
* β_2_*	NA	0.19 (0.10–0.30)
R_0_†	2.6 (2.0–4.1)	NA
R_0_ before	NA	8.72 (5.14–16.32)
R_0_ after	NA	1.33 (0.68–2.04)
R_0_ combined	NA	5.72 (3.62–8.70)
Intervention efficacy‡	NA	0.85 (0.66–0.94)
*t_init_*	−22 (−39 to −4)	−4 (−25 to −1)
AIC	657.33	628.85

*The value of *E_init_* was fixed at day 1 and the value of *t_inflect_* at day 12. The R_0_ values were calculated by using equations 4 and 5 (Appendix). AIC, Akaike information criterion; NA, not applicable; R_0_, basic reproduction number; *β*, current transmission rate per day; *β_1_*, transmission rate per day before inflection date; *β_2_*, transmission rate per day after inflection date; *E_init_*, number of initial infections at date *t_init_*; R_0_, basic reproduction number; *t_init_*, date on which the initial infection occurs. †R_0_ was calculated before and after inflection date in the 2-phase model. ‡The intervention efficacy was calculated as 1 – *β_2_*/*β_1_*. Days for *t_init_* are relative to the first positive sample on day 1.

**Figure 4:**
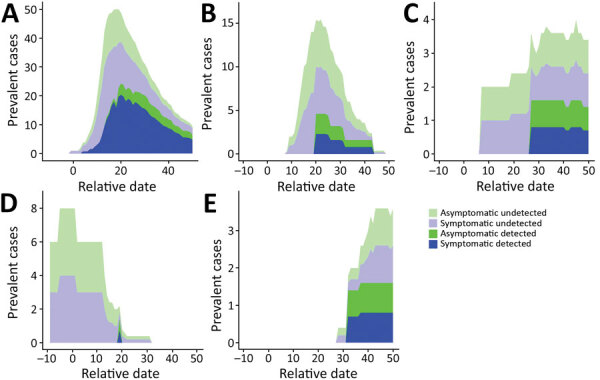
Stacked prevalence of detected and undetected symptomatic and asymptomatic infections in simulated epidemics using a model of nosocomial SARS-CoV-2 transmission determined on the basis of data from a long-term care facility in France. A) Prevalence estimated by using the 2-phase model for the whole hospital. B–E) Prevalence estimated by using the 1-phase model for individual wards: A2 (B), C0 (C), C2 (D), and C3 (E). After excluding extinct simulations (i.e., those having <3 cumulative cases), we calculated the median of each prevalence measure for each date.

In the 2-phase model where *t_inflect_* = 12, we observed a notable difference between the transmission rates estimated before and after *t_inflect_*, which we assume to be attributable to the new contact precautions. The transmission rate fell from 1.3 (95% CI 0.8–2.4) to 0.19 (95% CI 0.10–0.30) infections/patient/day in symptomatic infection, corresponding to a drop in R_0_ from 8.7 (95% CI 5.1–16.3) to 1.3 (95% CI 0.7–2.0). This result translates to an 85% (95% CI 66%–94%) decrease of the transmission risk after generalized implementation of contact precautions. Although the value of *t_inflect_* had a substantial effect on the absolute values of the transmission rates, the size of the decrease in transmission rate was relatively stable, ranging from 81%–89% (Appendix Table 4). At peak prevalence of infectious patients, we estimated the proportion of undetected infections at 60%, and overall, ≈25% of cases were undetected over the entire study period (Figure 4, panel A).

### Ward-Level Analysis

We calculated estimates and corresponding fits for each individual ward for which the 1-phase model could be validated (Table 2; Figure 3, panel B). We reconstructed the undetected parts of the epidemic (Figure 4, panel B). We also conducted ward-level analysis using the 2-phase model but this did not improve the fit (Appendix, Table 5). 

**Table 2:** Characteristics and parameter estimates in hospital wards in a long-term care facility in France used to estimate nosocomial transmission rates of SARS-CoV-2*

Ward	No. beds	Total no. patients	Day of first positive case	No. cases	*β*	R_0_†	*t_init_*
A2	48	62	11	30	1.29 (0.51–NE)	8.76 (3.47–NE)	2 (−14 to 29)
C0	37	74	16	22	0.56 (0.22–NE)	3.79 (1.50–NE)	4 (−39 to 9)
C2	37	48	7	15	2.13 (0.29–NE)	14.46 (1.97–NE)	−8 (−39 to –14)
C3	37	63	24	7	0.42 (0.11–1.30)	2.87 (0.75–8.84)	19 (−9 to 21)

*Estimates and 95% CI for *β*, R_0_, and *t_init_* are from the fitting the 1-phase model to data from each ward (*E_init_* = 1). In many instances, the upper bound of the 95% CI for *β*, and in the most likely value of *β* for some wards, could not be estimated due to a flat likelihood surface, in which case the value is given as NE. NE, not estimated; *β*, current transmission rate per day; *E_init_*, number of initial infections at date *t_init_*; R_0_, basic reproduction number; *t_init_*, date on which the initial infection occurs. †The R_0_ values were calculated using equation 4 (Appendix).

Point estimates for *β* ranged from 0.42 to 2.13 across the studied wards. We were only able to calculate an upper bound for the transmission rate in 1 ward, C3; the resulting range estimate of 0.42 (0.11–1.30) infections/patient/day corresponds to an R_0_ of 2.87 (0.75–8.84). However, we could estimate a lower bound for each ward; the highest value, 0.51 infections/patient/day in ward A2, corresponds to a minimum R_0_ of 3.47.

### Sensitivity Analysis Results

For most parameters, perturbing had relatively minor effects on the estimated transmission rates for the 2 phases, or on *t_init_* (Appendix, Figure 8). The transmission rate in the second phase, *β_2_*, was the most sensitive, and most markedly sensitive to the duration of symptomatic infection (1/*δ*).

### R_t_ Results

We calculated R_t_ estimates by using EpiEstim (Appendix, Figure 9). The value was initially 10, then fell to <3, before a second peak.

## Discussion

We developed a specific framework to analyze SARS-CoV-2 data from a hospital outbreak using a transmission model of patient-to-patient infection. We estimated transmission rates from a LTCF during March–April 2020, across the entire hospital and in individual wards. We assessed 1 or 2 phases of transmission delimited by a specific change date (*t_inflect_*) corresponding to implementation of contact precautions, including obligatory mask-wearing for patients and staff, and the cessation of group activities.

We found that the 2-phase model was better supported by the data aggregated across the entire hospital than a model with a single transmission rate, and the 2-phase model better captured the early peak in cases. Model validation suggested sufficient power to estimate transmission rates in 2 phases. The early phase rate (1.3 transmissions/patient/day) corresponded to an early R_0_ of 8.7 and the late phase rate (0.19 transmissions/patient/day) corresponded to a late R_0_ of 1.3. This change in transmission rate can largely be explained by the initial absence of preventive measures after the policy recommendation on day 6 and its gradual implementation over the next week. Under this assumption, the measures introduced were 85% (95% CI 66%–94%) effective at reducing transmission. The high estimates in the first phase suggest an explosive outbreak or superspreading event, which is consistent with the high secondary attack rate (33%). The estimates in the second phase, after the updated policy, might be more representative of current transmission rates in hospitals, which can provide and encourage the use of personal protective equipment.

Little research is available for the effect of contact precautions against SARS-CoV-2 transmission in healthcare settings. A meta-analysis of the effect of mask use against nosocomial transmission of coronaviruses found 67% protective efficacy of facemasks and 96% efficacy of N95 respirators (*11*), but the 1 study involving SARS-CoV-2 only examined a protective effect for healthcare workers (HCWs), which was unquantifiable because no infections were reported in the masked group (*12*). Several modeling studies have quantified the level of mask wearing that would prevent epidemic spread of SARS-CoV-2 in the community (*13*–*15*; D. Kai et al., unpub. data, https://arxiv.org/abs/2004.13553), but studies of interventions for prevention of patient-to-patient transmission in healthcare environments are lacking.

Few other studies have published estimates of R_0_ in healthcare settings. By analyzing the initial exponential growth phase of a hospital epidemic, one study computed an expedient estimate of R_0_ for patients (1.13) and hospital staff (1.21) (*16*), but that study did not account for asymptomatic infections and did not provide a range for the R_0_ estimates (*17*). In another study, the authors estimated an R_0_ of 1.021 (95% CI 1.018–1.024) across 12 nursing homes based on a single introduction per floor of each institution and a secondary attack rate of 4.1% among 930 residents (B. Reyné et al., unpub. data, https://doi.org/10.1101/2020.11.27.20239913). The heterogeneity of transmission between different wards was also demonstrated in a previous review and meta-analysis in which the authors calculated an average observed reproduction number of 1.18 across 4 different healthcare settings (*18*), but showed much heterogeneity between settings; 1 was 4.5, and 3 were <0.25. A fourth study analyzed several hospitals in Canada by using incident cases and estimated an R_0_ of 2.51, which ranged from 0.56 to 9.17 in individual facilities (*19*). However, the authors of that study did not model asymptomatic infection or account for negative test results or the outcomes of testing at different infectious stages (*19*).

To assess how estimates vary when looking at smaller subpopulations, we separately fit a 1-phase model to data from each ward. Using this method, we could not always estimate upper bounds of the transmission rates, probably because of strong stochasticity and scarcity of observed cases, an inherent feature of SARS-CoV-2 in which a large proportion of infected persons remain asymptomatic. However, our validation analyses suggested that point estimates for transmission rates across the wards could be consistently estimated. Applied to our dataset, estimated transmission rates ranged from 0.4 to 2.1, corresponding to an R_0_ of 2.9–14.5. This heterogeneity might have been driven by differences in the timing of and compliance with preventive measures or by differences in contact patterns between staff and patients.

Calibrating models to real hospital outbreaks and estimating transmission rates provides more realistic transmission models to evaluate scenarios with alternative surveillance or control measures. We estimated the response to introducing barrier interventions at the beginning of the COVID-19 pandemic, when population immunity was minimal. Investigating alternative scenarios involving contemporary levels of population immunity or other viral variants could be easily achieved by updating the model parameters, such as the initial level of immunity or transmission rates. Updating parameters would enable prediction of the probability and size of hospital outbreaks and evaluation of testing strategies to prevent spread. As mentioned, a major challenge in analyzing outbreaks in hospitals or other small, closed environments lies in the consideration of imperfect testing practice, which we addressed through the observation model. First, a substantial proportion of infectious persons were not symptomatic; therefore, they were less likely to be tested, and we accounted for this difference in the model testing policy. Second, PCR test sensitivity is imperfect and depends on the time from infection, which is we also reflected in our evolving test sensitivity for different stages of infection. Finally, testing procedures were not regular and might have been affected by many factors not directly related to the epidemiologic situation, such as the day of the week, the available testing capacity, or changing strategies at the local scale. We addressed irregular testing procedures by using the number of tests per day directly described in the data rather than determining the number of tests performed from the number of infected persons. The model also tracked testing status to include realistic probabilities for testing and retesting of patients.

We compared our results with R_t_ from the commonly used EpiEstim package, which demonstrated the additional value of our approach. Ignoring negative tests and the complexity of testing policies, this simpler approach captured the high initial R_0_ and subsequent fall but also showed a second peak that likely resulted from increased testing rather than an actual increase in transmission rate.

Our analysis has several limitations resulting from simplifying assumptions. First, we did not account for the possibility of imported infections other than the index case or cases; instead, we assumed that the force of infection from other patients would substantially outweigh that from the community. Second, because we had no data on infectious status for HCWs during the study period, we focused on patients and did not explicitly model acquisition by nor transmission from HCWs, although HCWs were implicitly considered potential vectors of patient-to-patient transmission. Rates of transmission from infectious patients to HCWs are relatively low (*20*,*21*), as are transmission rates from HCWs to patients (*22*), although these rates might have been higher in the early stages of the pandemic, considering low levels of hand hygiene (*23*). Ignoring the contribution of HCWs to new infections in the analysis suggests that we might have overestimated the transmission risk from infectious patients, but our estimates can still be interpreted as valid measures of the nosocomial risk to patients. Third, the model relies on parameters taken from the literature, which may be inaccurate. However, we conducted a sensitivity analysis to measure the sensitivity of transmission rates to appropriate variation in these parameters, and our main results remained unaffected. Finally, we note that the decision to analyze data from this hospital is partly due to the size of the outbreak, implying a selection bias toward a higher transmission rate than would be typical across all hospitals. However, >44,000 nosocomial infections were reported in France by February 14, 2021 (*24*), most of which consisted of clusters of cases; thus, our results can be interpreted as plausible for a hospital at risk for an outbreak. In addition, the model framework we propose is suitable for estimating transmission rates in any healthcare environment, and we provide some guidance for adaptation (Appendix).

In conclusion, the novel dynamic modeling framework we propose realistically simulates evolving testing policies and could easily be used on similar nosocomial COVID-19 datasets. The model also could be adapted for specific epidemiologic features, such as patient isolation. Overall, our results underline both the substantial potential effect of protective interventions introduced in healthcare settings and the considerable heterogeneity in transmission rates between hospital wards.

Appendix Additional information on a model to assess effects of R_0_ on nosocomial SARS-CoV-2 transmission.
