# Supplementary material for: Measuring Basic Reproduction Number to Assess Effects of Nonpharmaceutical Interventions on Nosocomial SARS-CoV-2 Transmission
Source: Emerg Infect Dis. 2022 Jul;28(7):1345–54. doi: 10.3201/eid2807.212339 (PMC9239897; doi:10.3201/eid2807.212339)
Supplement: Appendix — Additional information on a model to assess effects of R0 on nosocomial SARS-CoV-2 transmission. [file 21-2339-Techapp-s1.pdf]

Article DOI: https://doi.org/10.3201/eid2807.212339

Measuring Basic Reproduction Number to Assess Effects of Nonpharmaceutical Interventions on Nosocomial SARS-CoV-2 Transmission

Appendix

Additional Methods

Data Input and Parameter Estimation

SARS-CoV-2 RNA extraction was performed on a NucliSENS easyMAG (bioMérieux, https://www.biomerieux.com) device, strictly following the manufacturer's recommendations. Reverse transcription PCR (RT-PCR) was performed on an ABI QuantStudio 7 (Thermo Fisher Scientific, https://www.thermofisher.com) device, using the commercial RealStar SARS-CoV-2 RT-PCR Kit 1.0 (Altona Diagnostics, https://www.altona-diagnostics.com) test. Briefly, 10 μL of RNA is added to the 20 μL RT-PCR mix. Two targets are detected, one specific for betacoronavirus, and one specific for the SARS-COV-2 strains. Internal control was added in the lysis buffer to validate both extraction and amplification steps.

All patients were included in the study if they were in the hospital during the study period from day −10 to day 50. Daily data on tests, admissions, and discharges were input directly into the model from the hospital data (Appendix Figure 2). In most cases, patients were recorded as being in a particular ward, and these were used to create ward-specific datasets. In case of a gap in the patient record between recorded stays in different wards the transfer was assumed to occur at the midpoint of the gap. All data analyzed for this study at the whole hospital and ward levels, along with the R scripts used to conduct the analysis, are available at github.com/georgeshirreff/Hospital_R0_C19.

Parameters Estimated Directly from Longitudinal Hospital Data

The parameter $1/\omega$, the duration of the R_p stage during which persons who have recovered from infectious disease but continue to frequently test positive through PCR, was selected by calculating the likelihood of each duration according to the results of repeat tests. The data used were repeat tests taken after a patient had an earlier positive test (Appendix Figure 3, panel A). We assumed that the R_p stage began 7 days after the first positive test ($1/\delta$), the probability of testing positive during this stage was 30% (Z_{Rp}) and the probability of testing positive in the recovery (R) stage afterwards was 1% ($1 - \nu$) (Table 1). The likelihood reached a plateau where $1/\omega = 23$, so a value of 25 days was subsequently used as consistent with this result (Appendix Figure 3, panel B).

The parameter ϕ, the relative rate of retesting, has been crudely estimated from the data by counting the number of repeat tests, i.e., on persons who have been tested again without having symptoms develop (n = 211) divided by the sum of the number of first tests (n = 314) plus the number who were retested upon developing symptoms (n = 34), giving a retesting rate of $\phi = 60\%$. A bootstrap analysis was conducted on the dataset to estimate of 50% CI and 70% CI.

Hospital Prevention and Contact Policy

At the beginning of the study period, hospital policy did not specify the use of any masks during contact between healthcare workers (HCWs) and patients. Patients normally participated in group activities including leisure activities, meals, and joint physiotherapy sessions. Gloves and gowns were required by staff during any contact with bodily fluids. After March 17, 2020, the policy changed to require the wearing of surgical masks during proximity contact between HCWs and patients, and between staff members, as well as cancellation of all visits and group activities.

PCR testing, where capacity was available, was conducted on any patients with typical COVID-19 symptoms, namely persistent cough, fever, anosmia, or diarrhea. PCR was also conducted on any patients after suspected infectious contact with other patients, as well as on any patients being admitted to the hospital or moved between wards. Where capacity was lacking, patients displaying new symptoms were tested with priority. This testing procedure continued throughout the study period.

Mathematical Model

Observation Model and Differential Equations

We show the observational model that defines how testing and retesting is conducted for each stage of infection (Appendix Figure 1). We also show the structure of the transmission model (Figure 1). The observation model can be represented using differential equations that describe the change in state of each compartment in each time step (equation 1), in which $\lambda(t)$ is the force of infection (equation 2). The bold terms in equation 1 above refer to flows that are determined in part by the available data from a given day.

Equation 1

$$\frac{dS}{dt} = \mathbf{Admission}(\boldsymbol{t}) - \mathbf{Initiation}(\boldsymbol{S}, \boldsymbol{t}) - \lambda(t)S - \mathbf{Discharge}(\boldsymbol{S}, \boldsymbol{t}) - \mathbf{Test}(\boldsymbol{t}, \boldsymbol{S})$$

$$\frac{dS_T}{dt} = -\mathbf{Initiation}(\boldsymbol{t}, \boldsymbol{S_T}) - \lambda(t)S_T - \mathbf{Discharge}(\boldsymbol{t}, \boldsymbol{S_T}) + \mathbf{Test}(\boldsymbol{t}, \boldsymbol{S})$$

$$\frac{dE}{dt} = \mathbf{Initiation}(\boldsymbol{t}, \boldsymbol{S}) + \lambda(t)S - \alpha E - \mathbf{Discharge}(\boldsymbol{t}, \boldsymbol{E}) - \mathbf{Test}(\boldsymbol{t}, \boldsymbol{E})$$

$$\frac{dE_T}{dt} = \mathbf{Initiation}(\boldsymbol{t}, \boldsymbol{S_T}) + \lambda(t)S_T - \alpha E_T - \mathbf{Discharge}(\boldsymbol{t}, \boldsymbol{E_T}) + \mathbf{Test}(\boldsymbol{t}, \boldsymbol{E})$$

$$\frac{dE_a}{dt} = \alpha E(1-\psi) - E_a\gamma\kappa_2 - \mathbf{Discharge}(\boldsymbol{t}, \boldsymbol{E_a}) - \mathbf{Test}(\boldsymbol{t}, \boldsymbol{E_a})$$

$$\frac{dE_{aT}}{dt} = \alpha E_T(1-\psi) - E_{aT}\gamma\kappa_2 - \mathbf{Discharge}(\boldsymbol{t}, \boldsymbol{E_{aT}}) + \mathbf{Test}(\boldsymbol{t}, \boldsymbol{E_a})$$

$$\frac{dE_s}{dt} = \alpha E\psi - E_s\gamma - \mathbf{Discharge}(\boldsymbol{t}, \boldsymbol{E_s}) - \mathbf{Test}(\boldsymbol{t}, \boldsymbol{E_s})$$

$$\frac{dE_{sT}}{dt} = \alpha E_T\psi - E_{sT}\gamma - \mathbf{Discharge}(\boldsymbol{t}, \boldsymbol{E_{sT}}) + \mathbf{Test}(\boldsymbol{t}, \boldsymbol{E_s})$$

$$\frac{dI_a}{dt} = E_a\gamma\kappa_2 - I_a\delta\kappa_3 - \mathbf{Discharge}(\boldsymbol{t}, \boldsymbol{I_a}) - \mathbf{Test}(\boldsymbol{t}, \boldsymbol{I_a})$$

$$\frac{dI_{aT}}{dt} = E_{aT}\gamma\kappa_2 - I_{aT}\delta\kappa_3 - \mathbf{Discharge}(\boldsymbol{t}, \boldsymbol{I_{aT}}) + \mathbf{Test}(\boldsymbol{t}, \boldsymbol{I_a})$$

$$\frac{dI_s}{dt} = E_{sT}\gamma + E_s\gamma - I_s\delta - \mathbf{Discharge}(\boldsymbol{t}, \boldsymbol{I_s}) - \mathbf{Test}(\boldsymbol{t}, \boldsymbol{I_s})$$

$$\frac{dI_{sT}}{dt} = -I_{sT}\delta - \mathbf{Discharge}(\boldsymbol{t}, \boldsymbol{I_{sT}}) + \mathbf{Test}(\boldsymbol{t}, \boldsymbol{I_s})$$

$$\frac{dR_p}{dt} = I_s\delta + I_a\delta\kappa_3 - \omega R_p - \textbf{Discharge}(\boldsymbol{t}, \boldsymbol{R_p}) - \textbf{Test}(\boldsymbol{t}, \boldsymbol{R_p})$$

$$\frac{dR_{pT}}{dt} = I_{sT}\delta + I_{aT}\delta\kappa_3 - \omega R_{pT} - \textbf{Discharge}(\boldsymbol{t}, \boldsymbol{R_{pT}}) + \textbf{Test}(\boldsymbol{t}, \boldsymbol{R_p})$$

$$\frac{dR}{dt} = \omega R_p - \textbf{Discharge}(\boldsymbol{t}, \boldsymbol{R}) - \textbf{Test}(\boldsymbol{t}, \boldsymbol{R})$$

$$\frac{dR_T}{dt} = \omega R_{pT} - \textbf{Discharge}(\boldsymbol{t}, \boldsymbol{R_T}) + \textbf{Test}(\boldsymbol{t}, \boldsymbol{R})$$

Force of Infection and Basic Reproduction Number Calculation

The force of infection acting on susceptible patients (equation 2) is defined by the infectious populations, the transmission rate, β, and the total population size N (equation 3).

Equation 2

$$\lambda(t) = \frac{\beta\left(I_s + I_{sT} + \varepsilon(E_s + E_{sT}) + \kappa_1(I_a + I_{aT}) + \varepsilon\kappa_1(E_a + E_{aT})\right)}{N}$$

Equation 3

$$N = S + S_T + E + E_T + E_a + E_{aT} + E_s + E_{sT} + I_a + I_{aT} + I_s + I_{sT} + R_p + R_{pT} + R + R_T$$

The basic reproduction number (R_0) value of can be calculated directly from the parameters according to equation 4, which takes into account the full-blown symptomatic transmission rate (β), the probability of entering the symptomatic (ψ) or asymptomatic pathway ($1 - \psi$), the relative transmission rate of each stage of infection (ε, κ_1), and the rate of leaving each stage (γ and δ). In the 2-phase model, 2 R_0 values, $R_{0\ \text{before}}$ and $R_{0\ \text{after}}$, were calculated independently for each phase based on the different transmission rates, β_1 and β_2, using equation 4; a combined R_0 value was calculated as an average weighted by the duration of each phase using equation 5, with the final date being the end of the study period, day 50.

Equation 4

$$R_0 = \beta\left(\psi\left(\frac{\varepsilon}{\gamma} + \frac{1}{\delta}\right) + (1 - \psi)\kappa_1\left(\frac{\varepsilon}{\gamma} + \frac{1}{\delta}\right)\right)$$

Equation 5

$$R_{0\ \text{combined}} = \frac{R_{0\ \text{before}} \max\left(t_{inflect} - t_{init}, 0\right) + R_{0\ \text{after}} \left(\text{final\_date} - \max\left(t_{inflect}, t_{init}\right)\right)}{\text{final\_date} - \min\left(t_{inflect}, t_{init}\right)}$$

Deterministic Processes

The bold terms in equation 1 above refer to flows that are determined in part by the available data from a given day (d), including number of admissions $A(d)$, discharges $D(d)$, and of tests $T(d)$, as shown in weekly aggregate (Appendix Figure 2) or by specific parameter values, such as SARS-CoV-2 introduction date (t_{init}) and size (E_{init}), which together determine the number of daily new infectees $C(d)$, in the case of epidemic initiation. This calculation ensures that admissions, discharges, and number of tests each occur with the same frequency in the model as they do in the data, and that the timing and size of the start of the epidemic is determined by parameter values.

Admission

Admissions only occur into the susceptible untested (S) group, and so on a given day (d) the number of admissions into this group is exactly determined by the number of admissions on that day, $A(d)$:

Equation 6

$$\int_{t=d}^{t=d+1} \text{Admission}(t)dt = A(d)$$

Initiation

The expectation of the number of initial infectees from each susceptible group (S, S_T) is determined by equations 7 and 8, where $C(d)$ is equal to E_{init} on day t_{init}, and otherwise 0, with the total in both groups being equal to $C(d)$ (equation 10).

Equation 7

$$E\left(\text{Initiation}(d, S)\right) = C(d)\frac{S}{S + S_T}$$

Equation 8

$$E\left(\text{Initiation}(d, S_T)\right) = C(d)\frac{S_T}{S + S_T}$$

For simplification, we denote the number of infections initiated on day *d* in compartment *X* by equation 9, which is equal to the integral of all initiations in all (both) compartments across the whole of the day. The total initiations across all compartments is equal to *C*(*d*) (equation 10).

Equation 9

$$\text{Initiation}(d, X) = \int_{t=d}^{t=d+1} \text{Initiation}(t, X)dt$$

Equation 10

$$\sum\nolimits_{X \in S, S_T} \text{Initiation}(d, X) = C(d)$$

Discharge

The expectation of the number of discharges for a compartment *X* on each day (equation 11) has different values for symptomatically infected patients (I_s and I_{sT}), who have a discharge rate modified by the parameter μ. The denominator of the expectation, *W*, is the total dischargeable population, adjusting for these differences in rate (equation 12).

Equation 11

$$E\left(\text{Discharge}(d, X)\right) = \begin{cases} \frac{\mu X}{W} D(d) & \text{for } X \in I_S, I_{ST} \\ \frac{X}{W} D(d) & \text{for } X \notin I_S, I_{ST} \end{cases}$$

Equation 12

$$W = S + S_T + E + E_T + E_a + E_{aT} + E_s + E_{sT} + I_a + I_{aT} + \mu(I_s + I_{sT}) + R_p + R_{pT} + R + R_T$$

As with initiations above, we denote the number of patients discharged on day *d* in compartment *X* by equation 13, which is equal to the integral of all discharges in that compartment across the whole of the day. The total discharges on day *d* across all compartments *U* is equal to *D*(*d*) (equation 14).

Equation 13

$$\text{Discharge}(d, X) = \int_{t=d}^{t=d+1} \text{Discharge}(t, X)dt$$

Equation 14

$$\sum\nolimits_{X \in U} \text{Discharge}(d, X) = D(d)$$

Testing Model

The expectation of the number of tests to occur in a compartment *X* on day *d* is given as follows, with *T*(*d*) referring to the number of tests occurring on day *d* in the data (equation 15).

The untested symptomatic patients are tested as a priority and so the number of tests they receive is determined as the minimum of their size (I_s) and the number of tests available (*T*(*d*)), so the expected number of these (in equation 15 for I_s) is the same as their total (equation 18).

The remaining tests are distributed randomly throughout the remaining compartments. Expectations are shown in equation 15, derived from the number of tests left over after first symptomatic tests, the size of the compartment, the parameter φ for the already tested compartments, and a denominator *M* (equation 16) which represents the total testable population (including the adjustment for retesting).

Equation 15

$$E\big(\text{Test}(d, X)\big) = \begin{cases} \min(T(d), I_S) & \text{for } X \in I_S \\ \min(T(d) - I_S, 0)\dfrac{X}{M} & \text{for } X \in S, E, E_a, E_s, I_a, R_p, R \\ \min(T(d) - I_S, 0)\dfrac{\varphi X}{M} & \text{for } X \in S_T, E_T, E_{aT}, E_{sT}, I_{aT}, I_{sT}, R_{pT}, R_T \end{cases}$$

Equation 16

$$M = \big(S + E + E_a + E_s + I_a + R_p + R\big) + \varphi(S_T + E_T + E_{aT} + E_{sT} + I_{aT} + I_{sT} + R_{pT} + R_T)$$

As with initiations above, we denote the number of patients tested on day *d* in compartment *X* by equation 17, which is equal to the integral of all tests in that compartment across the whole of the day. The total tests across all compartments other than I_s are equal to the number of remaining tests (equation 19).

Equation 17

$$\text{Test}(d, X) = \int_{t=d}^{t=d+1} \text{Test}(t, X)dt$$

Equation 18

$$\text{Test}(d, I_S) = \min(T(d), I_S)$$

Equation 19

$$\sum\nolimits_{X \notin I_S} \text{Test}(d, X) = \min(T(d) - I_S, 0)$$

When simulating the observed tests on a given day using the function *rmeasure* in *pomp* (https://CRAN.R-project.org/package=pomp) for R (R Foundation for Statistical Computing, https://www.r-project.org), the number of positive and negative tests for a given day is drawn from the number of tests occurring in each compartment (equation 15) according to the probability that a sample taken from a patient in that compartment would test positive, which is governed by specificity, v, for virus-free compartments, and sensitivity z_X for each compartment X (equations 20 and 21). These expected distributions are also used for evaluating the likelihood of observed data using *dmeasure* in *pomp*.

Equation 20

$$\begin{aligned} E\big(\text{Positives}(d)\big) &= \big(\text{Test}(d, S) + \text{Test}(d, S_T)\big)(1 - v) + \big(\text{Test}(d, E) + \text{Test}(d, E_T)\big)z_E \\ &+ \big(\text{Test}(d, E_a) + \text{Test}(d, E_{aT})\big)z_{Ea} + \big(\text{Test}(d, E_s) + \text{Test}(d, E_{sT})\big)z_{Es} \\ &+ \big(\text{Test}(d, I_a) + \text{Test}(d, I_{aT})\big)z_{Ia} + \big(\text{Test}(d, I_s) + \text{Test}(d, I_{sT})\big)z_{Is} \\ &+ \Big(\text{Test}\big(d, R_p\big) + \text{Test}\big(d, R_{pT}\big)\Big) z_{Rp} + \big(\text{Test}(d, R) + \text{Test}(d, R_T)\big)(1 - v) \end{aligned}$$

Equation 21

$$E(\text{Negatives}(d)) = \big(\text{Test}(d,S) + \text{Test}(d,S_T)\big)v + \big(\text{Test}(d,E) + \text{Test}(d,E_T)\big)(1 - z_E) + \big(\text{Test}(d,E_a) + \text{Test}(d,E_{aT})\big)(1 - z_{Ea}) + \big(\text{Test}(d,E_s) + \text{Test}(d,E_{sT})\big)(1 - z_{Es}) + \big(\text{Test}(d,I_a) + \text{Test}(d,I_{aT})\big)(1 - z_{Ia}) + \big(\text{Test}(d,I_s) + \text{Test}(d,I_{sT})\big)(1 - z_{Is}) + \Big(\text{Test}\big(d,R_p\big) + \text{Test}\big(d,R_{pT}\big)\Big)\big(1 - z_{Rp}\big) + \big(\text{Test}(d,R) + \text{Test}(d,R_T)\big)v$$

Implementation of the Stochastic Model

The hybrid stochastic model was implemented by using the *rprocess* function in *pomp* using the Gillespie algorithm because the number of events on a given day is relatively small (around 15–30) given the population of <400 patients. The algorithm calculates a rate for each possible type of event to occur, determines the time and type of the next event accordingly, and then recalculates the rates after each event. To ensure that deterministic transitions (i.e., events determined by model input) occur with certainty within the framework of the Gillespie algorithm, the rate of such events was set to an arbitrarily large number, $L = 10^6$, if further instances of that event are still to occur on day *d*. Once all required instances have occurred, the rate is set to zero.

Statistical Inference

Likelihood Calculation

The infection model was linked to observed data (number of positive and negative tests per day) using the framework of a partially observed Markov process (POMP) in which the modified susceptible-exposed-infected-recovered (SEIR) model governs the underlying infection dynamics, and each day the observation process provides a likelihood of observing the data given the internal state.

The likelihood for the observation of several negative and positive tests on a particular day *d* is given in equation 22. The set of parameters is represented by θ, and normally only β and t_{init} would vary within the 1-phase model, but in the 2-phase model β_1, β_2, and t_{init} are estimated. The expected numbers of positives and negatives according to the model and θ are given by

equations 20 and 21. The total likelihood is the product of the likelihood values across all time points d.

Equation 22

$$\text{Likelihood}_d = p(\text{testing negative}(d)|\text{model}, \theta)^{\text{Negatives}(d)} p(\text{testing positive}(d)|\text{model}, \theta)^{\text{Positives}(d)}$$

$$= \left(\frac{E(\text{negatives}(d))}{\left(E(\text{negatives}(d)) + E(\text{positives}(d))\right)}\right)^{\text{Negatives}(d)} \left(\frac{E(\text{positives}(d))}{\left(E(\text{negatives}(d)) + E(\text{positives}(d))\right)}\right)^{\text{Positives}(d)}$$

Parameter Estimation through Stochastic Model Fitting

The inference of parameters (transmission rates β, or β_1 and β_2, and for t_{init} or E_{init}) was conducted according to the methodology proposed by King et al. (*6*). The first step was an initial search for the values of all parameters to be estimated, using 500 iterations of 500 particles, and a cooling fraction of 50% every 50 steps. This was repeated 10 times for each of 1,000 different starting points of the parameters to be estimated.

Subsequently a likelihood profile was estimated for β (in the 1-phase model) or β_1 (in the 2-phase model) by repeating the analysis above but using starting points for the parameter to be profiled across its relevant range (e.g., 0.1–10 in steps of 0.1), and holding this parameter constant while estimating the other(s) using the same inference methodology.

In all iterative filtering analyses, transmission rates were allowed to vary during an iteration, while t_{init} (or E_{init}) was only varied at the beginning of an iteration as an initial value parameter. During inference of the 2-phase model, each β-value was only allowed to vary during the phase in which it took direct effect, meaning β_1 would only vary before $t_{inflect}$ and β_2 would only vary afterwards.

Both the initial search and likelihood profiling were conducted using 500 iterations of 500 particles in each analysis, each of which was repeated 10 times for each of 1,000 different starting points of the parameters to be estimated.

The likelihood of the final parameter combination in each analysis (whether initial search or profiling) was then estimated by performing 10 repetitions of particle filtering with 100,000 particles, from which a linear average of the 10 likelihoods was taken.

Confidence Intervals for Estimated Parameters

Confidence intervals for estimated parameters were established by identifying sets of parameters values with a likelihood above a threshold relative to the highest likelihood for each analysis. The threshold was the maximum value of the likelihood minus half of the 95% quantile of the χ-square distribution with degrees of freedom corresponding to the number of parameters to be estimated, typically 2 for the 1-phase model (β and t_{init}) and 3 for the 2-phase model (β_1, β_2, and t_{init}).

Model Inference Validation

We conducted validation by using synthetic data to test the effectiveness of the model and statistical inference to recover known values of the parameters. Several datasets, representing numbers of “observed” positive cases, were generated from model simulations to represent the transmission within the whole hospital and the individual wards using known parameter values. The parameter values estimated from these datasets were then compared with the known values used to create them. Values of the parameters of interest were varied simultaneously, while all other parameters were fixed as depicted (Appendix Table 1).

Datasets were generated using *rmeasure* in *pomp* for each set of known parameter values over a 3-month observation period (day −39 to day 50). Multiple (n = 10) datasets were generated for each ward, or the whole hospital, and each set of known parameter values. In each case, the real data on numbers of daily tests, admissions, discharges, and number of patients were used. Iterative filtering to estimate the relevant parameters was then conducted on each dataset. The 1-phase model was validated by using data at the scale of both the whole-hospital and individual ward levels, while the 2-phase model was validated only at the whole-hospital level.

Unlike in the analysis on the true data, the value of β or β_1 was only estimated by iterative filtering directly, without the systematic likelihood profile for a range of values. For each parameter search, 500 iterations of 500 particles were used. For each known set of parameter

values, the median value of the estimated parameters was identified and compared with their known values.

To systematically identify wards with sufficient power to be analyzed using our inference methodology, the resulting estimated values were compared with true values. For each dataset, a deviation was estimated as the ratio of the estimated to the true value. If the median value of this deviation across all analyses for the ward was $\leq$1.15, it was considered that the ward had sufficient power to be analyzed.

Simulated Epidemic Curves

After identification of sets of parameter values with likelihoods within the 95% CI relative to maximum likelihood, these sets of parameter values were sampled with replacement 1,000 times, and each time an epidemic was simulated. Those parameters that went to extinction (having <3 cumulative infections) were excluded, and the remaining epidemics were used to calculate the median and 95% CI for relevant epidemic variables (number of positive tests, detected and undetected symptomatic and asymptomatic prevalence) for each date.

Due to repeat testing, it is not possible to calculate exactly the number of infections that were detected and undetected in simulated data, but equation 23 provides an approximation, where undetected includes both untested patients as well as false negatives. Symbols are described in Appendix Table 1.

Equation 23

Prevalence of undetected asymptomatics $= \left(E_a + I_a + E_{aT}(1 - Z_{Ea}) + I_{aT}(1 - Z_{Ia})\right)$

Prevalence of undetected symptomatics $= \left(E_s + I_s + E_{sT}(1 - Z_{Es}) + I_{sT}(1 - Z_{Is})\right)$

Prevalence of detected asymptomatics $= (E_{aT}Z_{Ea} + I_{aT}Z_{Ia})$

Prevalence of detected symptomatics $= (E_{sT}Z_{Es} + I_{sT}Z_{Is})$

Calculating Time-Varying Reproduction Number

We calculated the time-varying reproduction number, R_t, across the entire hospital and from the date of the first positive test until the 50th day, using the EpiEstim package

(https://CRAN.R-project.org/package=EpiEstim). The number of tests per day that were the first positive test for each patient was used as the daily incidence. We assumed a serial interval of 5.8 (range 4.8–6.8) days (*3*).

Considerations in Adapting to Alternative Scenarios

The model code is available on Github (github.com/georgeshirreff/Hospital_R0_C19) with the intention that this model can be applied to other healthcare environments. With minimal adjustment, the could be directly adapted to a dataset aggregating positive and negative tests for active infection, admissions, and discharges each day. It could easily be adapted to different types of SARS-CoV-2 tests by adjusting the sensitivity and specificity for different stages of infection. Testing for both active infection and serology could also be included with consideration of the outcomes from testing at each stage of infection and adjustment to the likelihood function to account for both testing streams.

Our model rests on an assumption of free mixing between persons, so ward-level analysis might be more appropriate where this is unrealistic. Simultaneous modeling of different subpopulations, such as HCWs and patients in different wards, could be straightforwardly achieved, requiring consideration of the relative contact rates between these groups. The number of iterations and particles used for the statistical inference, as well as the number of repetitions of the analysis, might need to be adjusted to the size of the dataset and the desired level of precision.

Supplementary Results

Validation of Statistical Inference of Model Parameters

Simultaneous estimation of known parameter values was conducted for both the 1- and 2-phase models on data at the scale of the whole hospital. We describe the relationship between known and estimated values of β and t_{init} for the 1-phase model (Appendix Figure 4). The value of β was well estimated throughout the range, but the estimate of t_{init} was slightly over-estimated from days −25 to −16, but not after this point.

We also describe the relationship for the 2-phase model between known values of β_1, β_2, and t_{init} and their estimates (Appendix Figure 5). The first phase transmission rate, β_1, was reliably estimated up to 1.0 with a slight overestimation after that point. The second phase

transmission rate, β_2, was correctly estimated, except for values wrongly estimated to be close to zero. However, in the absence of estimates close to zero in the analysis of true data, the estimates could be considered reliable. As with the 1-phase model, the estimate of t_{init} deviated slightly when it fell in the first weeks but was more reliable after day −15.

The ability of our framework to correctly estimate parameter values at the ward level was limited due to much smaller population sizes and specific distributions of tests. We illustrate the ability to estimate values of β and t_{init} simultaneously using the 1-phase model on individual wards (Appendix Figures 6,7). Our results suggest that only data corresponding to wards A2, C0, C1, C2, and C3 provided sufficient power to be analyzed through our framework, and C1 was also excluded due to the lack of visible relationship between true and estimated values.

Results of Whole-Hospital Analysis

We show results for all analyses of the 1-phase model (Appendix Table 2) and for $E_{init} = 1$ only (Table 1). We show results for all analyses of the 2-phase model with $t_{inflect}$ = day 12 (Appendix Table 3) and those for $E_{init} = 1$ only (Table 1). We also show analyses exploring the effect of changing $t_{inflect}$ (Appendix Table 4).

Results of Ward-Level Analysis

We compared results from each ward (Appendix Table 5). These results demonstrate that the Akaike information criterion (AIC) for the 1-phase model is lower or equal for 3 of 4 wards.

Sensitivity Analysis

We calculated best estimates and 95% CI for β_1, β_2, and t_{init} (Appendix Figure 8). Many parameters affect the upper ranges of β_1, most markedly ε which also affects the best estimate. However, the relative effects on β_2 are much greater; δ and the sensitivity parameters of form Z_x have a large effect on both the mean estimate and range. An early inflection point, $t_{inflect}$, serves to suggest the possibility of an earlier epidemic initiation point, t_{init}, but the biggest effect on t_{init} comes from perturbing the number of index cases, E_{init}, with a larger number of indices pointing to a later introduction.

Time-Varying Reproduction Number

The estimated time-varying reproduction number (R_t) had an initially high value, 10 (1.8–23.7), which is consistent with our own estimate of R_0 in the first phase (Appendix Figure 9). R_t drops to a low of 2.8 (0.7–5.8), which reflects the fall in our own estimate in the second

phase of the analysis, representing a decrease of 72%. A similar magnitude is observed by calculating the mean R_t before, 8.0 (1.5–18.9), and after, 2.1 (1.3–3.1), our estimated change point, $t_{inflect}$. The analysis also displays a second peak after the first fall in R_t. Because this method only accounts for incident cases and ignores the evolving testing rate, it is likely that this peak reflects that substantial increase in testing rate rather than a true increase in transmission rate.

References

1. Buitrago-Garcia D, Egli-Gany D, Counotte MJ, Hossmann S, Imeri H, Ipekci AM, et al. Occurrence and transmission potential of asymptomatic and presymptomatic SARS-CoV-2 infections: A living systematic review and meta-analysis. PLoS Med. 2020;17:e1003346. PubMed https://doi.org/10.1371/journal.pmed.1003346

2. Li R, Pei S, Chen B, Song Y, Zhang T, Yang W, et al. Substantial undocumented infection facilitates the rapid dissemination of novel coronavirus (SARS-CoV-2). Science. 2020;368:489–93. PubMed https://doi.org/10.1126/science.abb3221

3. He X, Lau EHY, Wu P, Deng X, Wang J, Hao X, et al. Temporal dynamics in viral shedding and transmissibility of COVID-19. Nat Med. 2020;26:672–5. PubMed https://doi.org/10.1038/s41591-020-0869-5

4. Kucirka LM, Lauer SA, Laeyendecker O, Boon D, Lessler J. Variation in false-negative rate of reverse transcriptase polymerase chain reaction-based SARS-CoV-2 tests by time since exposure. Ann Intern Med. 2020;173:262–7. PubMed https://doi.org/10.7326/M20-1495

5. Ra SH, Lim JS, Kim G, Kim MJ, Jung J, Kim S-H. Upper respiratory viral load in asymptomatic individuals and mildly symptomatic patients with SARS-CoV-2 infection. Thorax. 2021;76:61–3. PubMed https://doi.org/10.1136/thoraxjnl-2020-215042

6. King AA, Nguyen D, Ionides EL. Statistical inference for partially observed Markov processes via the R package pomp. J Stat Softw. 2016;69:1–43. https://doi.org/10.18637/jss.v069.i12

Appendix Table 1. Symbols, parameters, values, and state variables used in a model of basic reproduction number of nosocomial SARS-CoV-2 transmission

Symbol	Parameter	Value (95% CI)	Source
t_{init}	Date on which the initial infection occurs	Estimated from the modeling analysis	NA
$t_{inflect}$	Date on which the value of β changes in the 2-phase model	Estimated within range 1–16 (relative to date of first positive sample)	NA
E_{init}	The number of initial infections at date t_{init}	1 (default), 3 or 10 cases	NA
β_1	Transmission rate per day before the inflection date	Estimated from the modeling analysis	
β_2	Transmission rate per day after the inflection date	Estimated from the modeling analysis	NA
β	The current transmission rate per day, or the single transmission rate in the 1-phase model	Estimated from the modeling analysis	
ε	Relative transmission rate from during pre-symptomatic infection compared with symptomatic infection	0.63 (0.18–2.26)	(*1*)
$1/\alpha$	Mean duration of non-infectious incubation in days	3.4 (3.3–4.0)	(*2*) Latent period
$1/\gamma$	Mean duration of infectious incubation stage in days	2.3 (0.8–3.0)	(*3*) Duration of pre-symptomatic infection
$1/\delta$	Mean duration of full-blown infection in days	7 (2.4–9.1)	(*3*) with uncertainty proportional to that of duration of infectious incubation
$1/\omega$	Mean duration of viral shedding following recovery from infectious stage in days	20 (0–60)	Hospital data; see Appendix Methods; Appendix Figure 3
Ψ	Proportion entering symptomatic pathway	0.69 (0.62–0.76)	(*1*)
κ_1	Relative infectivity of asymptomatics in full infection relative to full symptomatic infection	0.35 (0.1–1.27)	(*1*)
κ_2, κ_3	Relative rates of progression to full infection (E_a to I_a) and recovery in asymptomatic pathway, relative to symptomatic pathway	1	Assumption
μ	Relative rate of discharge for symptomatic patients relative to any non-symptomatic patient	1	Assumption
Z_E, Z_{Ea}, Z_{Es}, Z_{Ia}, Z_{Is}, Z_{Rp}	PCR test sensitivity for E, E_a, E_s, I_a, I_s, or R_p states	0.1 (0–0.5), 0.7, 0.7 (0.25–0.85), 0.8, 0.8 (0.65–0.9), 0.3 (0.2–0.5)	(*4*) with (*5*) confirming that viral loads in symptomatic and asymptomatic infection are similar
v	PCR test specificity	0.99 (0.96–0.992)	Assumption (lower bound of range comes from A.N. Cohen et al., unpub. data, http://medrxiv.org/lookup/doi/10.1101/2020.04.26.20080911)
φ	Relative rate of retesting compared to testing for the first time	0.6 (0.5–0.7)	Hospital data; see Appendix Methods
$A(d)$	The number of admissions in the data on day d		Hospital data
$D(d)$	The number of discharges in the data on day d		Hospital data
$T(d)$	The number of tests in the data on day d		Hospital data
$C(d)$	The number of infections initiated (moved from S to E or S_T to E_T on day d)		t_{init} and E_{init}
$\lambda(t)$	Force of infection at time t		State variable
			State variable
W	The total rate adjusted number of dischargeable individuals across all compartments at a given time		State variable
M	The total rate adjusted number of testable individuals across all compartments including those eligible for retesting, but excluding untested I_s, at a given time		State variable
N	The total population size at a given time		State variable
U	The universal set of compartments		State variable
Admission(*t*)	Admissions occurring into compartment S at time t		State variable

Initiation(*X*,*t*)	Infection initiations occurring from compartment *X* at time *t*	State variable
Discharge(*X*,*t*)	Discharges occurring from compartment *X* at time *t*	State variable
Test(*X*,*t*)	Tests occurring from compartment *X* at time *t*	State variable
S	Susceptible untested at a given time	State variable
E	Infected uninfectious untested at a given time	State variable
E_a	Early infectious infection on asymptomatic pathway, untested at a given time	State variable
E_s	Pre-symptomatic infectious infection on symptomatic pathway, untested at a given time	State variable
I_a	Full-blown infection on asymptomatic pathway, untested at a given time	State variable
I_s	Full-blown symptomatic infection, untested at a given time	State variable
R_p	Recovered but still shedding virus, untested at a given time	State variable
R	Recovered and no longer shedding virus, untested at a given time	State variable
S_T	Susceptible tested at a given time	State variable
E_T	Infected uninfectious tested at a given time	State variable
E_{aT}	Early infectious infection on asymptomatic pathway, tested at a given time	State variable
E_{sT}	Pre-symptomatic infectious infection on symptomatic pathway, tested at a given time	State variable
I_{aT}	Full-blown infection on asymptomatic pathway, tested at a given time	State variable
I_{sT}	Full-blown symptomatic infection, tested at a given time	State variable
R_{pT}	Recovered but still shedding virus, tested at a given time	State variable
R_T	Recovered and no longer shedding virus, tested at a given time	State variable

Appendix Table 2. Best estimates and the ranges for β in the 1-phase model and corresponding R_0 values to assess nosocomial SARS-CoV-2 transmission*

Estimate	β	R_0	E_{init}	t_{init}†	AIC
β, t_{init}	0.38 (0.30–0.60)	2.6 (2.0- 4.1)	**1**	−22 (−39 to −4)	657.3257
	0.40 (0.29–0.62)	2.7 (2.0- 4.2)	**3**	−8 (−38 to −2)	656.5639
	0.38 (0.26–0.60)	2.6 (1.8- 4.1)	**10**	−4 (−11 to 0)	653.7993
β, E_{init}	0.37 (0.27–0.61)	2.5 (1.8- 4.1)	2.7 (1.5–19.9)	**−6**	654.4575
	0.37 (0.26–0.61)	2.5 (1.8- 4.1)	2.5 (0.5–11.3)	**−13**	656.2111
	0.40 (0.29–0.57)	2.7 (2.0- 3.9)	1.8 (0.5- 7.8)	**−20**	655.3993

*R_0 values are calculated using equation 4. Bold text indicates fixed values. AIC, Akaike information criterion; NE, not estimated; β_1, transmission rate per day before the inflection date; β_2, transmission rate per day after the inflection date; E_{init}, number of initial infections at date initial infection occurs; R_0, basic reproduction number; t_{init}, date initial infection occurs.
†Values for t_{init} are relative to the day of the first positive sample.

Appendix Table 3. Best estimates and their ranges for β_1, β_2 from the 2-phase model and corresponding R_0 values to assess nosocomial SARS-CoV-2 transmission*

Estimate	E_{init}	β_1	β_2	$R_{0\ before}$	$R_{0\ after}$	t_{init}	AIC
β_1, β_2, t_{init}	**1**	1.28 (0.76–2.40)	0.19 (0.10–0.30)	8.72 (5.14–16.32)	1.33 (0.68–2.04)	−4 (−24 to 0)	628.85
	**3**	1.23 (0.68–2.20)	0.19 (0.10–0.28)	8.37 (4.65–14.96)	1.31 (0.66–1.89)	−2 (−13 to 4)	628.4
	**10**	1.03 (0.59–2.80)	0.20 (0.10–0.27)	7.03 (4.00–19.04)	1.39 (0.67–1.85)	0 (−6 to 6)	631.21
β_1, β_2, E_{init}	0.70 (0.50–2.49)	1.10 (0.63–2.00)	0.20 (0.11–0.30)	7.48 (4.26–13.60)	1.39 (0.76–2.02)	**−6**	634.51
	0.63 (0.50–2.43)	1.14 (0.69–2.10)	0.20 (0.10–0.29)	7.78 (4.68–14.28)	1.36 (0.69–1.96)	**−13**	631.74
	1.12 (0.50–7.47)	1.24 (0.70–2.20)	0.19 (0.10–0.29)	8.42 (4.73–14.96)	1.32 (0.67–1.95)	**−20**	629.24

*R_0 values were calculated using equation 4, substituting the corresponding β value. Bold text indicates fixed values. AIC, Akaike information criterion; NE, not estimated; β_1, transmission rate per day before the inflection date; β_2, transmission rate per day after the inflection date; E_{init}, number of initial infections at date initial infection occurs; R_0, basic reproduction number; t_{init}, date initial infection occurs.

Appendix Table 4. Effect of changing $t_{inflect}$ on estimated parameter values in the 2-phase model to assess nosocomial SARS-CoV-2 transmission*

$t_{inflect}$	β_1	β_2	$R_{0\ before}$	$R_{0\ after}$	$R_{0\ combined}$†	Intervention efficacy	t_{init}	AIC
1	3.44 (0.29–49.50)	0.36 (0.23–0.57)	23.38 (2.00–336.67)	2.47 (1.60–3.88)	13.59 (2.41–180.07)	0.89 (−0.47 to 0.99)	−4 (−37 to 1)	654.27
6	2.68 (0.99–49.50)	0.29 (0.17–0.40)	18.21 (6.71–336.67)	1.98 (1.16–2.70)	10.61 (4.50–179.87)	0.89 (0.64–1.00)	−2 (−19 to 6)	639.97
8	2.24 (0.99–12.10)	0.25 (0.16–0.34)	15.22 (6.73–82.30)	1.68 (1.07–2.35)	8.87 (4.39–44.43)	0.89 (0.68–0.98)	−2 (−20 to 3)	633.56
10	1.62 (0.91–4.00)	0.22 (0.14–0.32)	11.01 (6.18–27.21)	1.49 (0.96–2.14)	6.55 (4.14–15.12)	0.86 (0.67–0.95)	−3 (−22 to 1)	629.89
12	1.28 (0.76–2.00)	0.19 (0.10–0.30)	8.72 (5.14–13.60)	1.33 (0.68–2.04)	5.26 (3.38–7.94)	0.85 (0.66–0.94)	−4 (−24 to 1)	628.85
14	1.02 (0.70–1.50)	0.17 (0.08–0.26)	6.91 (4.73–10.20)	1.15 (0.56–1.77)	4.22 (3.06–6.09)	0.83 (0.63–0.94)	−5 (−27 to 2)	630.19
16	0.84 (0.54–1.08)	0.16 (0.07–0.26)	5.69 (3.65–7.31)	1.07 (0.48–1.78)	3.53 (2.61–4.29)	0.81 (0.61–0.93)	−5 (−28 to 2)	634.83

*Best estimates and ranges for β_1, β_2, corresponding R_0 values, and t_{init}. R_0 values are calculated using equation 4, substituting the corresponding β value. The risk ratio is calculated for each point estimate as β_1/β_2. AIC, Akaike information criterion; β_1, transmission rate per day before the inflection date; β_2, transmission rate per day after the inflection date; R_0, basic reproduction number; $t_{inflect}$, date on which the value of β changes in the 2-phase model; t_{init}, date initial infection occurs.
†The combined R_0 is an average R_0 in each phase weighted by phase duration as in equation 5.

Appendix Table 5. Best estimates and ranges for β_1, β_2, and R_0 in each phase, combined, and t_{init} for each hospital ward in a 2-phase model to assess nosocomial SARS-CoV-2 transmission*

	2-phase, value (95% CI)†						1-phase
Ward	β_1	β_2	Risk ratio‡	$R_{0\ combined}$§	t_{init}	AIC	AIC
A2	2.16 (0.30–NE)	0.70 (0.31–4.42)	0.33 (0.04–11.39)	10.41 (4.77–49.01)	4 (−20 to 7)	139.4	138.25
C0	NE	0.35 (0.26–4.89)	0.04 (0.03–7.20)	39.44 (1.75–50.23)	10 (−38 to 11)	89.91	91.59
C2	NE	0.00 (0.00–0.08)	0.00 (0.00–0.06)	37.25 (1.16–38.52)	−14 (−33 to −12)	57.62	57.92
C3	6.50 (0.00–NE)	0.41 (0.23–0.64)	0.06 (0.03–NE)	26.43 (1.03–39.82)	20 (−26 to 21)	47.25	45.25

*The values of E_{init} was fixed at day 1 and $t_{inflect}$ was fixed at day 11. AIC, Akaike information criterion; NE, not estimated; β_1, transmission rate per day before the inflection date; β_2, transmission rate per day after the inflection date; E_{init}, number of initial infections at date initial infection occurs; R_0, basic reproduction number; $t_{inflect}$, date on which the value of β changes in the 2-phase model; t_{init}, date initial infection occurs.
†In many instances, the upper bound of the 95% CI for β_1, and in some also the most likely value of β_1, could not be estimated due to a flat likelihood surface, in which case the value is given as NE.
‡The risk ratio is calculated for each point estimate as β_1/β_2.
§R_0 values were calculated using equations 4 and 5.

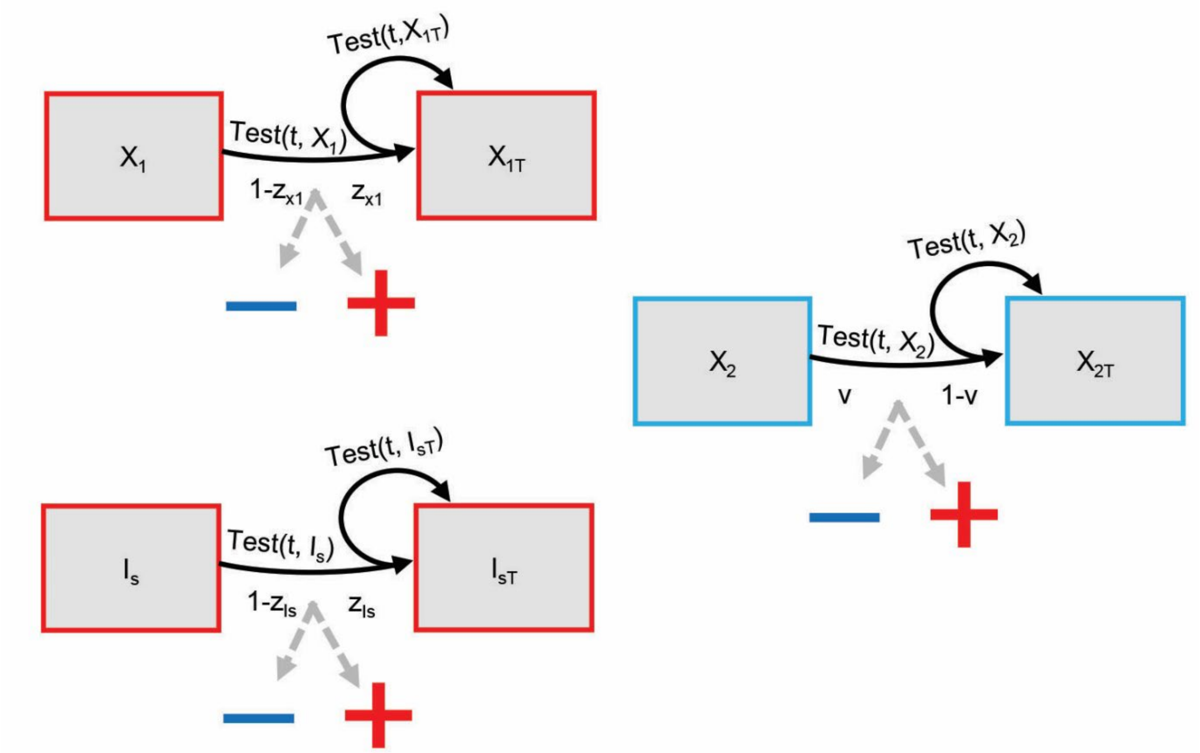

Appendix Figure 1. Illustration of the observation process for a model of nosocomial SARS-CoV-2 transmission. X_1 represents any compartment of untested persons who are shedding virus (E, E_a, E_s, I_a, R_p) who test positive at their compartment-specific sensitivity rate (z_{X1}), and X_2 represents any compartment of persons not recently tested who are not shedding virus (S, R) who test negative at rate v. X_{1T}, I_{sT}, and X_{2T} represent tested counterparts. The symptomatic persons (I_s and I_{sT}) are shown separately because testing is conducted first on the non-recently tested symptomatic group, but retesting is equally likely for symptomatic persons as for asymptomatic persons. Upon testing or retesting, the dotted arrows indicate the probabilities of the possible observed outcomes, positive and negative. E, exposed; E_a, asymptomatic exposed; E_s, symptomatic exposed; I_a, asymptomatic infected; I_s, symptomatic infected; I_{sT}, symptomatic infected and tested; R, recovered; R_p, recovered but shedding virus; S, susceptible; +, positive; – negative.

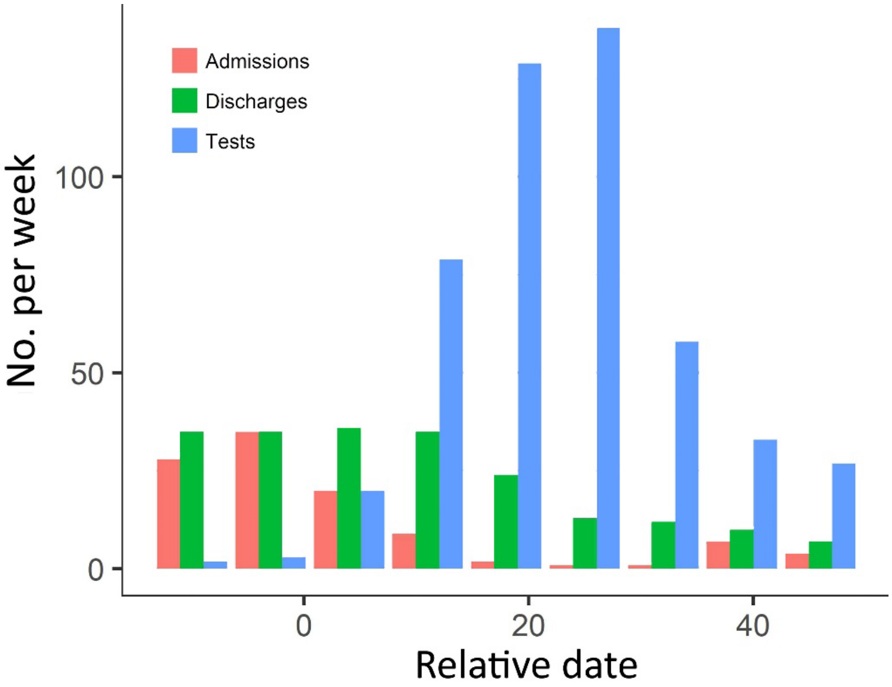

Appendix Figure 2. Weekly aggregated number of admissions, discharges, and PCR tests reported over the study periodin a hospital used for developing a model of basic reproduction number of nosocomial SARS-CoV-2 transmission. The daily disaggregated data are used in the model as *A*(*d*), admissions/day; *D*(*d*), discharges/day; and *T*(*d*), tests/day.

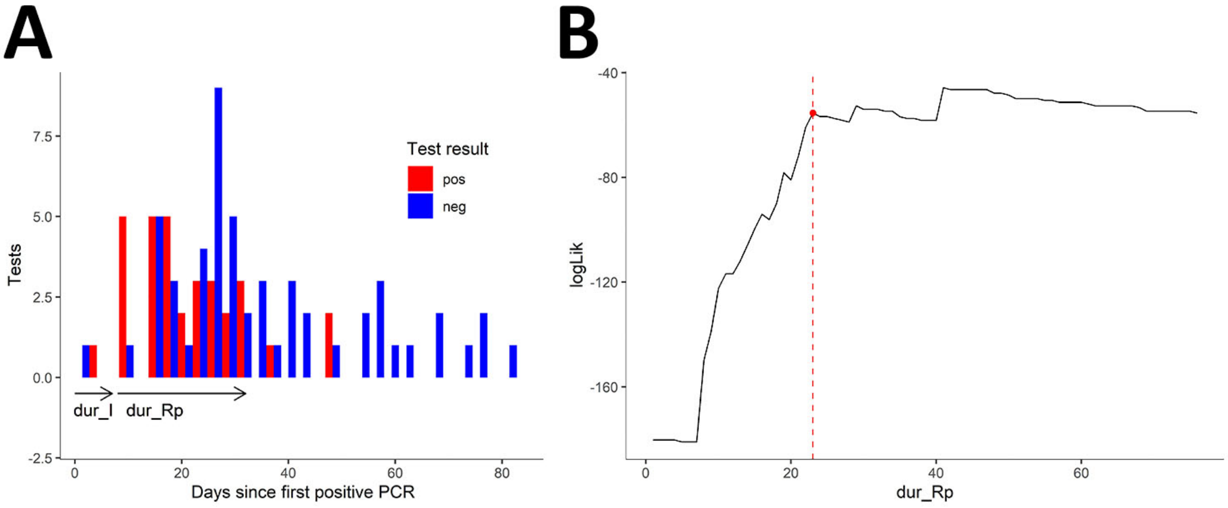

Appendix Figure 3. Results from repeat tests taken after a first PCR–positive test among patients in a hospital used to develop a model of basic reproduction number of nosocomial SARS-CoV-2 transmission. A) Number of positive (pos) and negative (neg) PCR tests reported. The duration of the full-blown infection stage was 7 days (Table 1). B) Likelihood for each potential duration (in days) of the R_p stage according to the sensitivity of the subsequent stages and the number of tests from each. dur_I, duration of infectiousness; dur_R_p, duration of viral shedding after recovered; R_p, recovered but shedding virus.

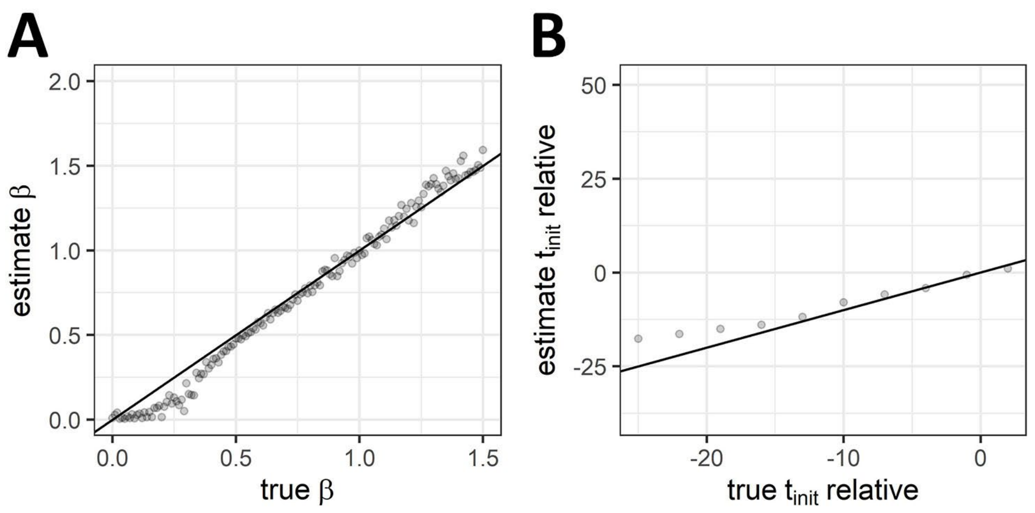

Appendix Figure 4. Validation of simultaneous estimation of transmission rate and initial infection date using the 1-phase model of nosocomial SARS-CoV-2 transmission on datasets at the whole-hospital level. A) Estimation of β. B) Estimation of t_{init}. Each point represents a true value of the parameter on its x-axis, with the value on the y-axis being the median across 10 attempts to estimate the true value using particle filtering. The solid black line indicates where the true and estimated values are equal. The value of E_{init} was fixed at 1. E_{init}, number of initial infections at date initial infection occurs; t_{init}, date initial infection occurs; β, current transmission rate per day.

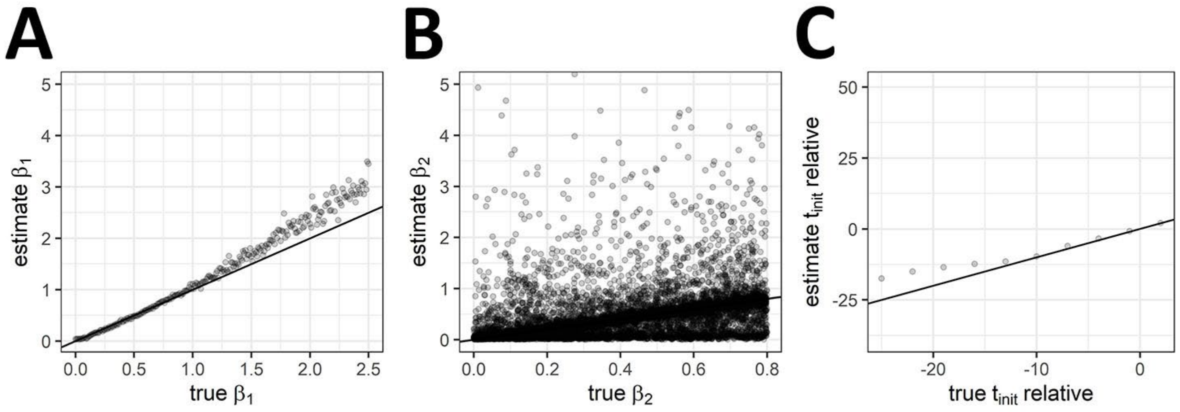

Appendix Figure 5. Validation of simultaneous estimations of transmission rates and initial infection date using a 2-phase model of nosocomial SARS-CoV-2 transmission on the datasets at the scale of the whole hospital. A) Validation for β_1. B) Validation for β_2. C) Validation for t_{init}. Each point represents a true value of the parameter on its x-axis; the value on the y-axis is the median across 10 attempts to estimate the true value using particle filtering. Values for E_{init} were fixed at day 1 and values for $t_{inflect}$ were fixed at day 12. Solid black line indicates where the true and estimated values are equal. E_{init}, number of initial infections on the date initial infection occurs; $t_{inflect}$, date on which the value of β changes in the 2-phase model; t_{init}, date initial infection occurs; β_1, transmission rate per day before the inflection date; β_2, transmission rate per day after the inflection date.

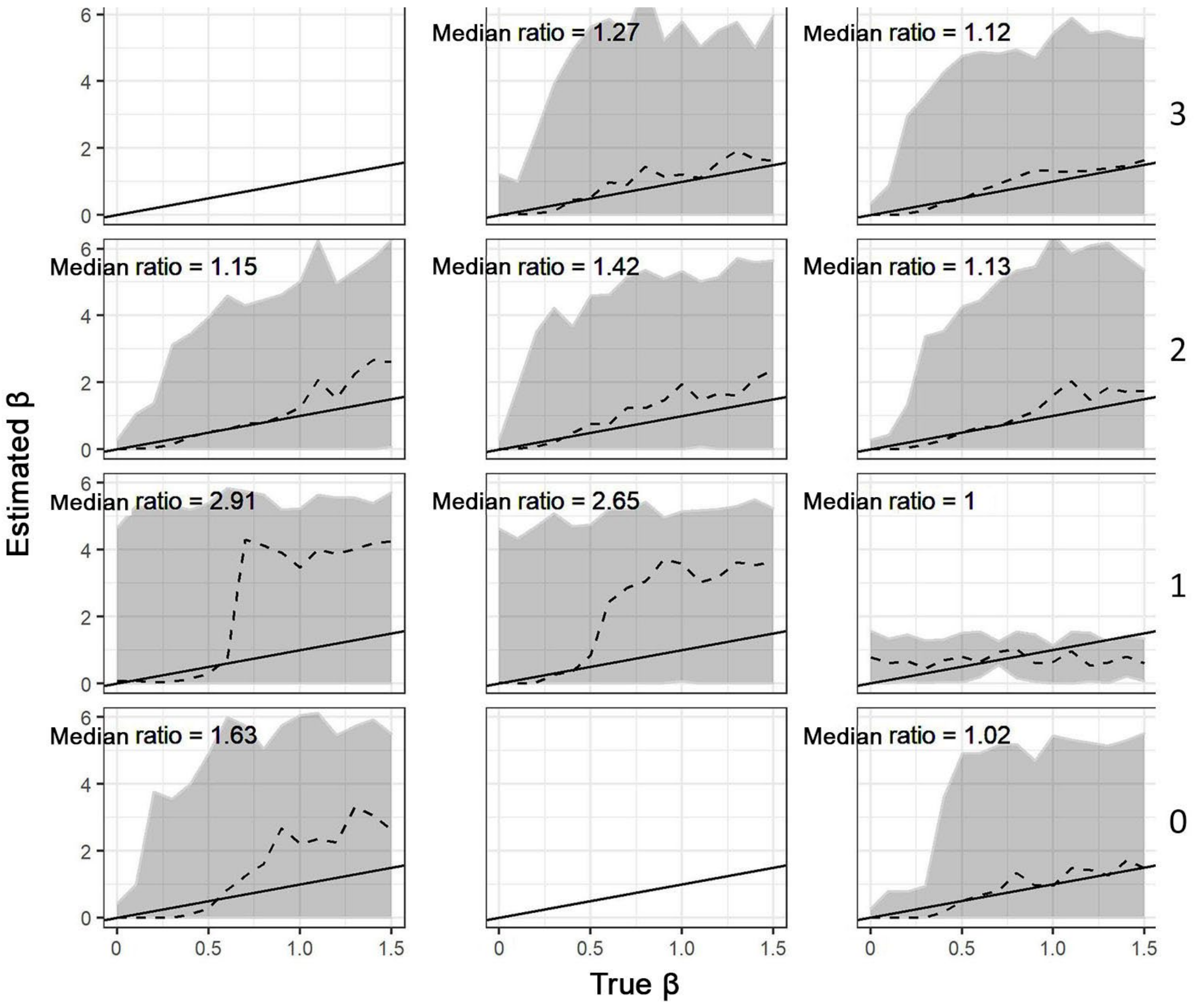

Appendix Figure 6. Validation of the estimation of *β* using the 1-phase model of nosocomial SARS-CoV-2 transmission on datasets at the ward level in a hospital used for developing a model to measure basic reproduction number. Columns represent the hospital buildings A–C (left–right); rows 0–3 represent the floors in each building. The black dashed line represents the median estimate on the y-axis for each true value of the parameter on the x-axis. The gray area represents the 95% range of estimates for each value of the true parameter. The solid black line indicates where the true and estimated values are equal. The value of E_{init} was fixed at 1. The numerical value given in the corner is the median ratio between the estimated and true values. E_{init}, number of initial infections at date initial infection occurs; *β*, current transmission rate per day.

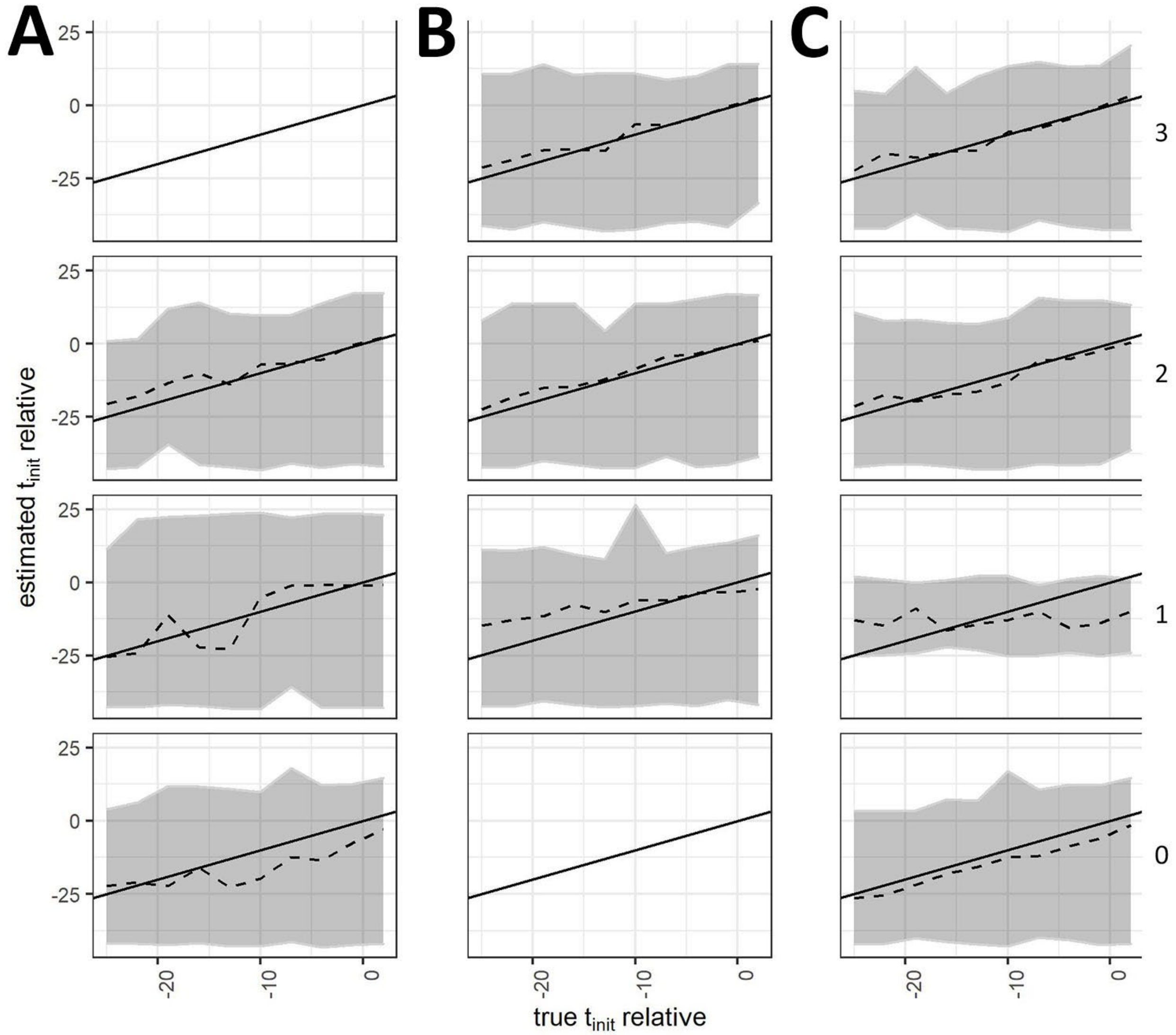

Appendix Figure 7. Validation of the estimation of initial infection date (t_{init}) using the 1-phase model of nosocomial SARS-CoV-2 transmission on datasets at the ward level. Columns A–C represent the hospital buildings; rows 0–3 represent the floors in each building. The black dashed line represents the median estimate on the y-axis for each true value of the parameter on the x-axis. The gray area represents the 95% range of estimates for each value of the true parameter. The solid black line indicates where the true and estimated values are equal. The value of E_{init} was fixed at 1. E_{init}, number of initial infections at date initial infection occurs.

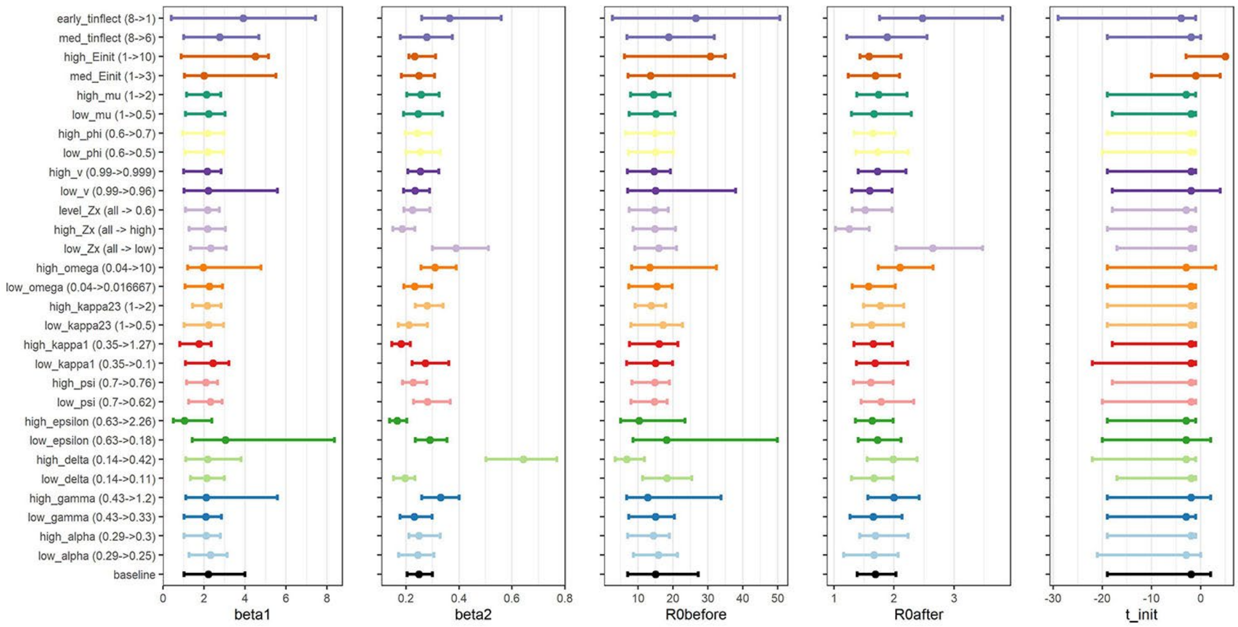

Appendix Figure 8. Results of sensitivity analysis of a 2-phase model of nosocomial SARS-CoV-2 transmission. Analysis shows beta1 (β_1), beta2 (β_2), and their corresponding R_0 values $R_{0before}$ (corresponds to β_1), and R_{0after} (corresponds to β_2); and t_{init} under parameter values perturbed according to their uncertainty ranges (Table 1; Appendix Methods), with all other parameters held at baseline values. Error bars indicate 95% CI, the corresponding dot shows the values of the parameter values that had the highest likelihood. In the scenario modifying Z_x, all Z_x parameters were modified simultaneously, to their lower or upper bounds, or to 0.6. In the scenario kappa23, both κ_2, the relative rates of progression from stage E_a, and κ_3, the relative rate of progression from stage I_a, compared to the equivalent symptomatic stage, were modified by the same factor. R_0, basic reproduction number; E_a, asymptomatic exposed; E_{init}, number of initial infections at date initial infection occurs; I_a, asymptomatic infected; $t_{inflect}$, date on which the value of β changes in the 2-phase model; t_{init}, date initial infection occurs; β_1, transmission rate per day before the inflection date; β_2, transmission rate per day after the inflection date, Z_x, PCR test sensitivity.

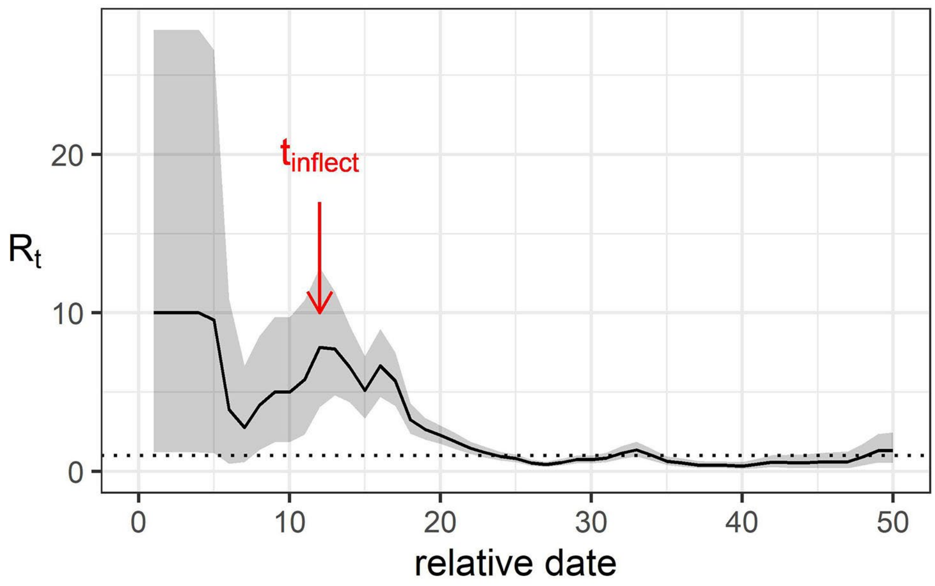

Appendix Figure 9. Time-varying reproduction number of nosocomial SARS-CoV-2 transmission. Estimation performed by using the EpiEstim package (https://CRAN.R-project.org/package=EpiEstim), and on the basis of incident cases, using a serial interval mean of 5.8 days and standard deviation of 0.51. The solid black line indicates the median estimate. The gray area indicates the 95% credibility interval. The red arrow indicates our best estimate for the transmission rate change point in our 2-phase model analysis. $t_{inflect}$, date on which the value of β changes in the 2-phase model; β, current transmission rate per day.
